# Airway Management for Tracheoesophageal Fistula Repair and Tracheal Repair Under Veno-Venous Extracorporeal Membrane Oxygenation

**DOI:** 10.7759/cureus.88903

**Published:** 2025-07-28

**Authors:** Ian En Koh, Suhitharan Thangavelautham, Harikrishnan Kothandan

**Affiliations:** 1 Anesthesiology, Singapore General Hospital, Singapore, SGP

**Keywords:** advanced airway management, endobronchial intubation, one-lung ventilation (olv), thoracotomy, three-field esophagectomy, tracheal stenosis, tracheoesophageal fistula repair, veno-venous extracorporeal membrane oxygenation (vv ecmo)

## Abstract

Tracheoesophageal fistula (TEF) resection and tracheal repair are complex, multidisciplinary operations requiring careful planning and consideration for a safe and successful procedure. In particular, airway management and maintaining oxygenation are of vital importance. We report a 54-year-old female with a history of pulmonary tuberculosis complicated by tracheal stenosis, which required multiple bronchoscopic interventions. She developed a TEF, resulting in respiratory failure requiring veno-venous extracorporeal membrane oxygenation (VV-ECMO) support for her airway interventions. She underwent tracheoesophageal resection and tracheal repair. Airway control was secured with bronchoscopic-guided endobronchial intubation and was subsequently re-positioned postoperatively, precisely within a narrow margin of safety, to maintain adequate ventilation yet avoid pressure on the tracheal repair. This case highlights the considerations and challenges regarding airway management in a patient with severe tracheal stenosis and TEF and demonstrates the utility of VV-ECMO in high-risk airway interventions.

## Introduction

Tracheal stenosis, a pathological narrowing of the trachea, often leads to significant respiratory distress and is commonly caused by post-intubation injury, infections, or inflammation. Tuberculosis (TB) is a recognized cause, particularly in regions with high TB prevalence. Tracheobronchial involvement in TB occurs in up to 40% of cases, with the reported incidence of airway stenosis developing in as many as 6%-50% [1]. Tracheoesophageal fistula (TEF) is an abnormal connection between the trachea and esophagus, often causing severe pulmonary complications due to aspiration. While congenital TEFs are well-documented, acquired forms are uncommon, linked to malignancy, infection, or trauma. They most frequently result from malignancies - particularly esophageal or lung cancers - with approximately 5% to 15% of patients with esophageal cancer, and about 1% of those with bronchogenic carcinoma, developing TEF [2]. Stent erosion causing a TEF is a rare but significant complication of tracheal stenting, bypassing laryngeal protection and leading to aspiration and respiratory compromise [3].

This report discusses a case with a protracted history of tracheal stenosis secondary to pulmonary TB, further complicated by tracheal stent erosion into the esophagus, resulting in a TEF. We highlight the airway management and the difficulties encountered during her therapeutic interventions.

## Case presentation

A 54-year-old lady presented to the Emergency Department (ED) with acute shortness of breath (SOB). She has a significant past medical history of pulmonary TB in 1998, which was fully treated at the time. However, this was complicated by tracheal stenosis, for which she had multiple bronchoscopic interventions, including ablation of granulation tissue, injection of triamcinolone, as well as balloon dilation and stent insertions. In the past, she was reviewed by a thoracic surgeon for tracheal surgery; however, this was not recommended in view of the high risks. She was on regular follow-up with Respiratory Medicine (RES). Her latest intervention occurred about a month ago, when her stent was found to be 80% occluded by sloughy granulation tissue. This was ablated and debrided. Topical mesna and adrenaline were applied.

**Figure 1:**
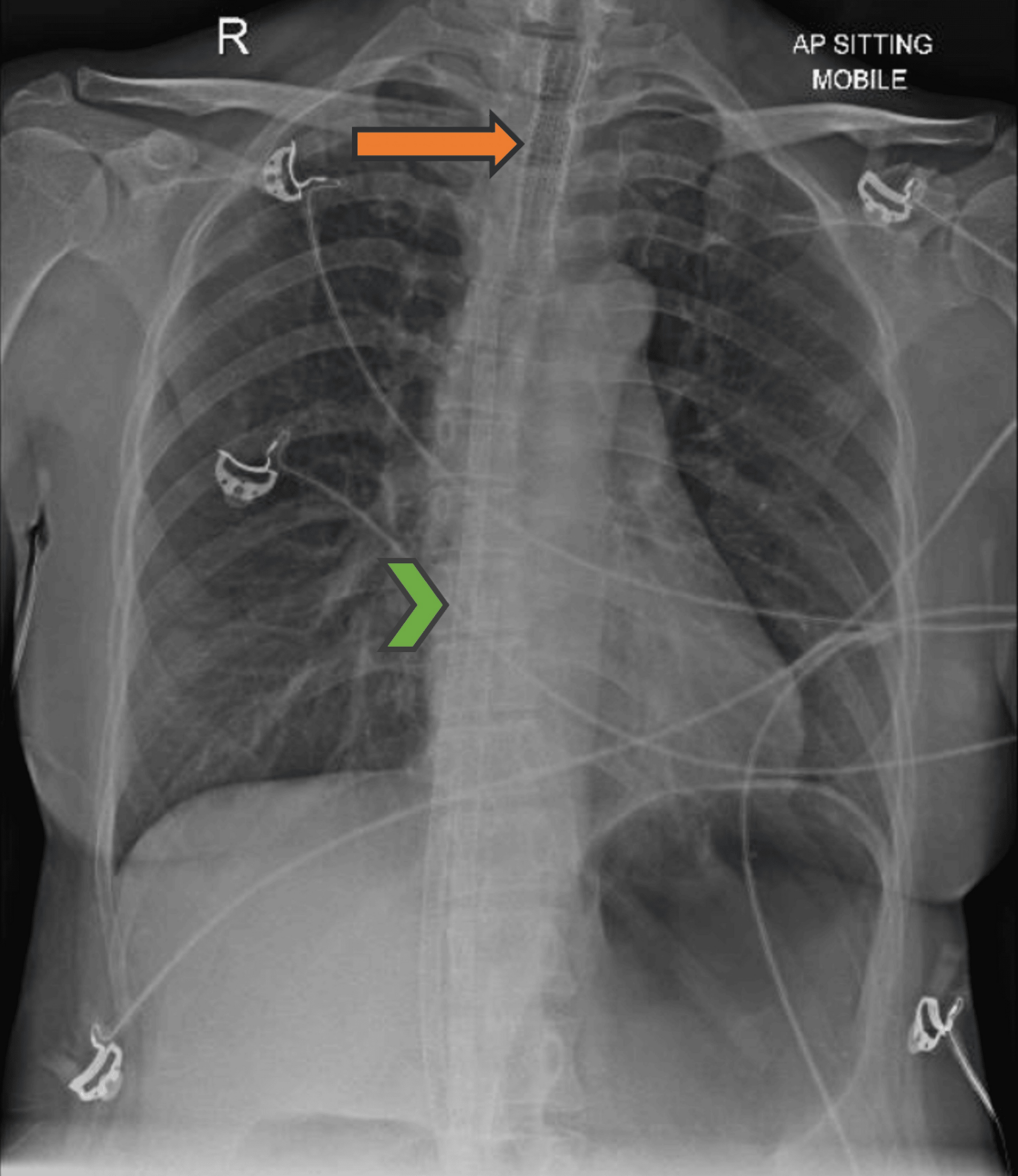
Preoperative chest X-ray showing tracheal stent (orange arrow) and VV-ECMO catheters (green chevron). VV-ECMO, veno-venous extracorporeal membrane oxygenation

In the ED, she was noted to be in respiratory distress with stridor. She was afebrile and was not able to expectorate sputum. On examination, her oxygen saturation was 93%-94% on facemask oxygen supplementation at 6 L/min, respiratory rate (RR) 20/minute, heart rate (HR) 100-130/minute, and blood pressure (BP) 147/82 mmHg. She used writing to communicate and was in a tripod position. Arterial blood gas on initial presentation was: pH 7.327, PaCO₂ 46.2 mmHg, PaO₂ 178.7 mmHg, base excess -2.6. The chest X-ray was not able to visualize the tip of the tracheal stent well, and there was no focal consolidation or sizable pleural effusion (Figure 1).

She was given three cycles of nebulized adrenaline and intravenous (IV) dexamethasone 8 mg, and IV glycopyrrolate 200 mcg. The patient’s condition improved after the second cycle of nebulized adrenaline. She was reviewed by a multidisciplinary team comprised of RES, Anesthesia (ANA) Airway, and Medical Intensive Care Unit (ICU) specialists. They were unable to perform a flexible nasendoscopic airway examination, as the patient was in acute distress. She was initiated on a trial of non-invasive ventilation (NIV) with the following settings: BiPAP iPAP 10, ePAP 5, FiO₂ 0.21, maintaining oxygen saturations of 95%-96%.

She was admitted to the Medical ICU with a working diagnosis of tracheal stenosis with recurrent stent obstruction and was planned for emergency rigid bronchoscopy and tracheal stenting. However, in view of the high risks of airway occlusion during the procedure - leading to inadequate ventilation and hypoxia - veno-venous extracorporeal membrane oxygenation (VV-ECMO) was initiated before rigid bronchoscopy. Oxygen saturations were 100% with VV-ECMO support (ECMO flow: 4.13 L/min, oxygen/air flow: 3 L/min, FiO₂: 1.0).

**Figure 2:**
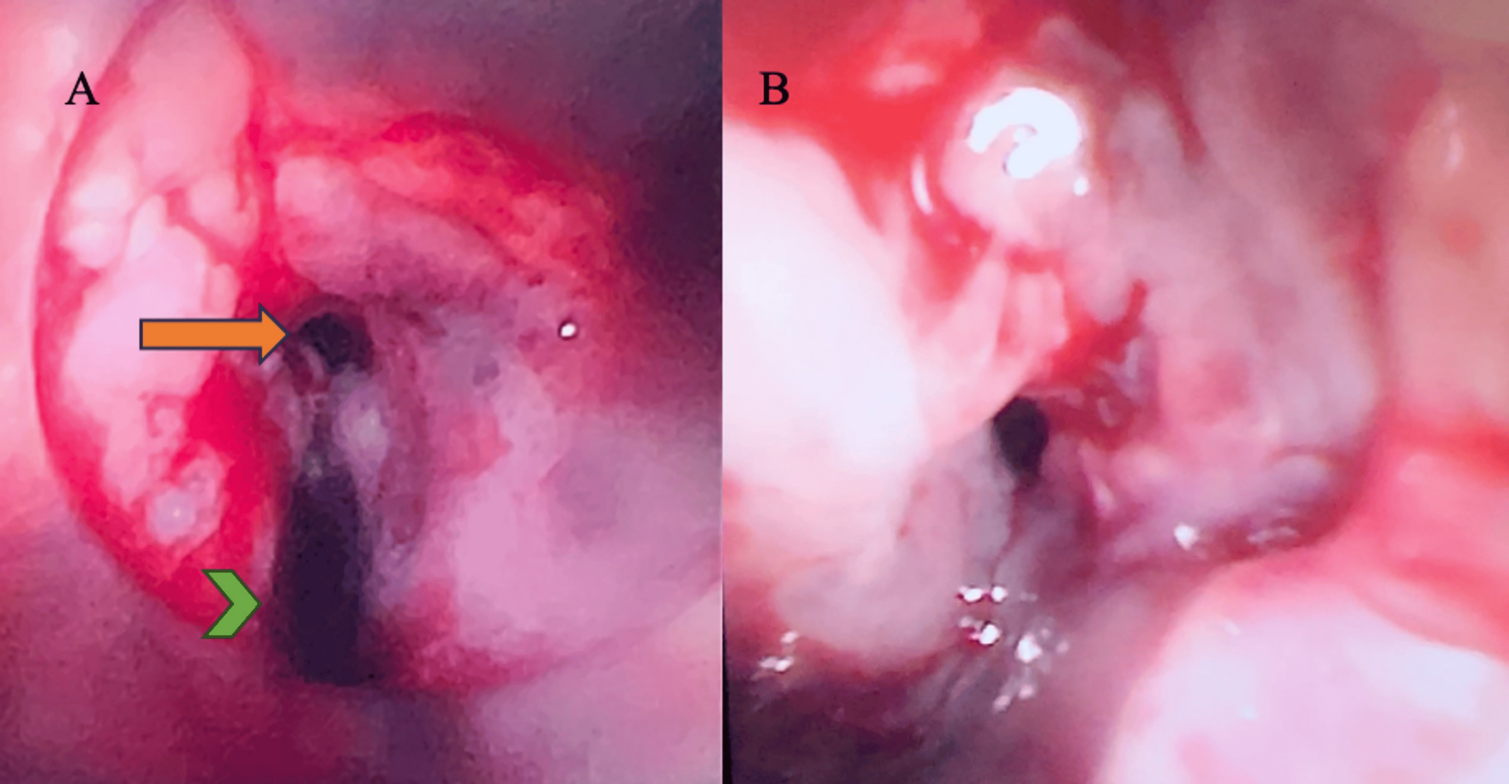
(A) Trachea (orange arrow) and tracheoesophageal defect (green chevron) on bronchoscopy; (B) tracheal stenosis.

The patient underwent emergency rigid bronchoscopy, removal of the tracheal silicone stent, and insertion of interlocking Ultraflex stents. A large TEF (indicated by the green chevron in Figure 2A) was identified about 40 mm from the carina. The size of the defect was 20-30 mm. Interlocked Ultraflex stents (14 × 60 mm + 16 × 60 mm; Boston Scientific Corporation, Natick, MA, USA) were deployed over the defect, bridging the TEF and re-establishing her airway. The patient was extubated and breathing normally postoperatively.

In view that the defect would not resolve nor heal well with conservative management, there was a multidisciplinary discussion between the Upper Gastrointestinal (UGI) Surgical, Cardiothoracic (CTS), Head and Neck (HNN), RES, and ANA teams, and the patient was planned for a combined surgery with VV-ECMO support as follows: rigid bronchoscopy and removal of tracheal stents; bronchoscopic-guided intubation by RES; transcervical access to the cervical esophagus by HNN; minimally invasive esophagectomy (three-field) and substernal gastric pull-up, feeding jejunostomy by UGI; and right thoracotomy and repair of the tracheal defect by CTS.

The following airway plan was discussed and agreed upon at the multidisciplinary discussion: we would first attempt videolaryngoscopy and intubation with an armored size 7.0 endotracheal tube (ETT), and isolate the right lung with a bronchial blocker (Plan A). Failing this, we would perform left endobronchial intubation (Plan B). Plan C was a surgical tracheostomy. If we were unable to intubate with a size 7.0 ETT, then the plan was to downsize the ETT and intubate endobronchially. If crossfield ventilation was required, CTS would perform a tube exchange with an armored ETT.

The patient was transferred to the operating room with VV-ECMO. A quad-lumen central venous catheter was inserted in the right internal jugular vein pre-induction. VV-ECMO support was increased to full support, with sweep gas at 4 L/min, up from 0.5 L/min. Thereafter, she was pre-oxygenated, induced with IV propofol, and maintained on total intravenous anesthesia (TIVA) with propofol and remifentanil infusions.

RES took over her airway and commenced rigid bronchoscopy. During this time, the patient was apnoeic, and gas exchange was sustained on VV-ECMO. The tracheal stents were removed, and the TEF was examined. The trachea was noted to be edematous, and a TEF was identified, with a tracheal defect of 20-30 mm located 40 mm above the carina (Figure 2A). There was chronic proximal tracheal stenosis, with inflamed, narrowed airways extending 30-40 mm (Figure 2B). Another smaller posterior tracheal wall defect, 10 mm distal to the fistula, was observed. A third pinhole posterior defect was noted 20 mm proximal to the carina intraoperatively. In discussion with CTS, RES, and ANA, a decision was made for bronchoscopic-guided left endobronchial intubation with a size 7.0 armored ETT to secure the airway beyond the level of the defects. The ETT position was confirmed clinically and bronchoscopically. Subsequently, the patient was turned to the left lateral position, and surgery commenced.

After completion of the thoracic phase, the patient was turned supine for the abdominal and cervical phases. The trachea was repaired via the left neck approach with remnant healthy esophageal tissue and surgical sutures, and subsequently via the right thoracotomy approach with surgical sutures. An intercostal muscle flap was harvested and used to buttress the repair posteriorly.

The tracheal repair underwent an underwater leak test in the supine position. The ETT was retracted past the repair site, guided by CTS, and gentle positive pressure was exerted above the level of the repair. There was no obvious air leak on the underwater check. However, the patient was not able to be ventilated through the ETT thereafter, with high airway pressures and low tidal volumes recorded. Bronchoscopic guidance was used to reposition the ETT. This was challenging due to copious amounts of secretions from the operative field, a narrowed airway due to edema, as well as altered anatomy from the primary pathology and surgical manipulation. Simultaneously, the margin of error for ETT placement was very small: 5 mm too deep would result in endobronchial intubation; 10 mm proximal would result in pressure over the TEF repair site. During this time, the patient was not ventilated and relied on full VV-ECMO support.

**Figure 3:**
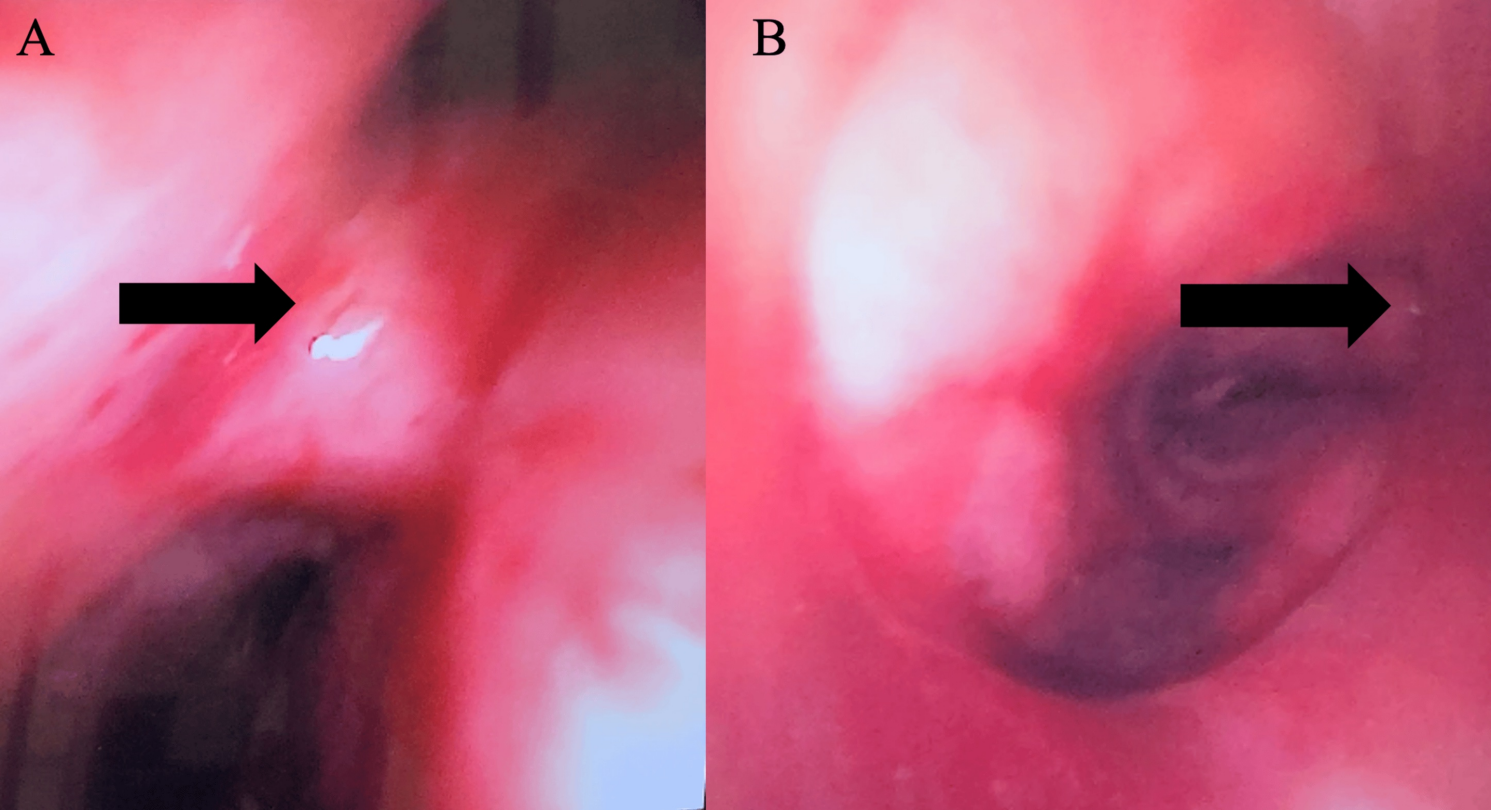
(A) Tracheal carina and left main-stem bronchus; carina marked by arrow. (B) Final position of ETT adjusted post-operatively with bronchoscopic guidance; carina marked by arrow. ETT, Endotracheal Tube

**Figure 4:**
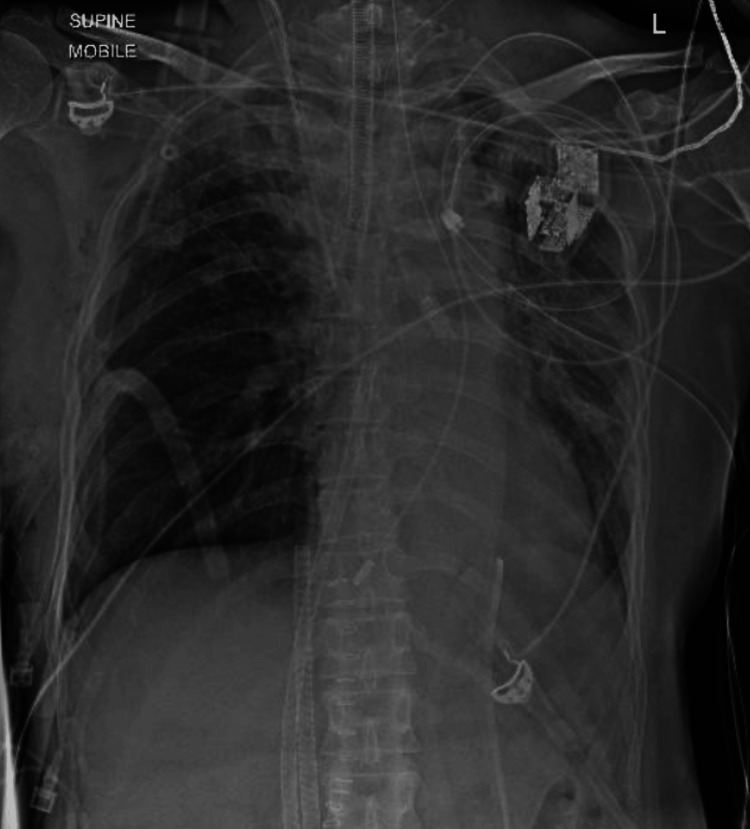
Post-operative chest X-ray showing the final position of the armored ETT. ETT, Endotracheal Tube

The size 7.0 armored ETT was guided to its final post-operative position, just above the carina, to ventilate bilateral lungs (Figures 3A-3B). The cuff was left deflated to avoid local trauma to the tracheal repair. Figure 4 shows the final armored ETT position on chest X-ray. Post-operatively, the patient was transferred to the cardiothoracic surgical ICU for continued ventilatory monitoring and ECMO support. 

On post-operative day (POD) 8, the patient underwent scheduled bronchoscopy, tracheal toileting, and cervical tracheostomy creation. Bronchoscopy through the existing armored ETT was difficult, as the lumen had narrowed due to biting by the patient, despite the use of a bite guard. The armored ETT was removed, and a laryngeal mask airway (LMA) was inserted, through which bronchoscopy was performed. Intra-operatively, the patient was noted to have upper tracheal stenosis involving 50% of the cross-sectional area, and lower tracheal stenosis that was 80%-90% narrowed by posterior invagination of the repair/muscular membrane, extending from the pre-existing tracheal stenosis to the carina. There were copious amounts of hemoserous exudates in the airway. Finally, CTS and RES decided that a cervical tracheostomy should be created to maintain airway patency, facilitate VV-ECMO weaning, and allow for airway toileting. A cervical tracheostomy was created, and an adjustable flange size 7.0 tracheostomy tube was inserted to stent the long-segment tracheal narrowing.

On POD 10 of tracheal repair, VV-ECMO support was weaned while the patient was on pressure support ventilation (pressure support 10 cm H₂O, positive end-expiratory pressure (PEEP) 5 cm H₂O, FiO₂ 0.4). The patient was able to maintain saturations of 98%-100% and remained hemodynamically stable. On POD 11, she was taken off ventilatory support and placed on a tracheostomy mask. Subsequently, on POD 23 of tracheal repair, she was stepped down from the ICU to high dependency.

## Discussion

This was a case of a patient with a complex airway history, long-standing tracheal stenosis, and TEF secondary to stent erosion, requiring surgical repair of her tracheal defect and esophagectomy for her TEF in a multidisciplinary, combined operation.

Airway management

Securing the airway in a patient with simultaneous TEF and tracheal stenosis undergoing esophagectomy, gastric pull-up, and tracheal repair presents a unique challenge. There was a combination of fibrosis from previous interventions, edema from recent therapeutics, resulting in significant airway narrowing, necessitating the use of downsized airway instruments. The airway tissues were weak and vulnerable, increasing the risk of trauma during intubation or surgical manipulation. Furthermore, ventilation with positive-pressure ventilation post-intubation could be challenging due to air leak via the TEF. VV-ECMO was initiated in our patient before the rigid bronchoscopy and tracheal stent placement, which avoided all the above challenges.

Securing the airway in a patient on VV-ECMO was a vital step for several practical reasons. It helps protect the lungs from contamination by blood, secretions, or gastric contents, reducing the risk of complications like aspiration pneumonia. Another key factor is maintaining lung recruitment, which is crucial even when ECMO is handling gas exchange. By securing the airway, we can use ventilation strategies that keep the lungs open and reduce the risk of lung injury. It also allows for controlled ventilation, which allows us to monitor end-tidal carbon dioxide levels and avoid inadvertent respiratory acidosis. In this case, we were able to use the ETT to perform checks for air leaks and ensure the surgical repair was holding. Lastly, having the airway secured ensures a fallback option for emergencies, such as an ECMO malfunction or sudden deterioration, where rapid ventilation and oxygenation could be lifesaving. This proactive approach ensures the patient remains as stable as possible while supported by ECMO.

This case involved a significant neck dissection to mobilize structures to facilitate gastric pull-up and tracheal repair, which risked recurrent laryngeal nerve (RLN) injury. The use of a nerve integrity monitoring (NIM) ETT may be indicated to facilitate real-time monitoring of the RLN, minimizing the risk of nerve injury that could result in vocal cord paralysis or dysphonia. Proper placement of the NIM tube is critical, as incorrect positioning may compromise the reliability of intraoperative neuromonitoring [4]. However, to secure the airway beyond the level of the defect, the electrodes of the NIM tube would not be in contact with the vocal cords. Although it may have been useful, the NIM tube could not be used for this case.

During lower tracheal or carinal surgeries, cross-field ventilation may be necessary. This involves retracting the oral endobronchial tube and intubating the bronchus across the operative field, and allows ventilation while the surgeon repairs the defect around the carina [5]. In this case, the initial plan of tracheal resection was changed to a tracheal repair and did not require cross-field ventilation.

In this case, there were multiple considerations to keep the patient intubated postoperatively. These included the edematous tracheal mucosa, airway narrowing, and a negative leak test, all indicating a high risk of airway compromise. The underlying tracheal stenosis, and the presence of airway secretions and blood, further increased the risk of obstruction. A prolonged surgery presents the likelihood of airway edema and impaired reflexes, necessitating airway protection and controlled ventilation. The patient was maintained on propofol and fentanyl infusions postoperatively to prevent coughing or agitation that could disrupt the tracheal repair.

TIVA during VV-ECMO

ECMO significantly alters the pharmacokinetics of TIVA agents, requiring increased doses due to hemodilution and drug sequestration in the ECMO circuit - the latter particularly in cases of lipophilic drugs such as propofol and fentanyl [6]. Target-controlled infusion models assume a fixed volume of distribution and do not take into account sequestration, potentially leading to inadequate dosing of anesthetic agents and an increased risk of awareness. We used continuous propofol (mg/h) and remifentanil (mcg/kg/min) infusions with manual dose titrations throughout. Nonetheless, close monitoring is vital due to the unpredictable pharmacodynamic response. Multiple endpoints should be monitored, including hemodynamic changes from sympathetic stimulation, patient reaction to surgical stimuli, and depth of anesthesia monitors such as Bispectral Index (BIS). Intra-operatively, VV-ECMO was monitored by a dedicated perfusionist. Blood flows were titrated at 3.5 to 4 L/min; sweep gas flows were at 3.5 to 4 L/min. PaCO_2_ was maintained between 35.2 and 47 mmHg intraoperatively.

## Conclusions

TEF secondary to tracheal stent erosion is an uncommon condition. Patients who present with recurrent aspiration pneumonia, with a history of prior TB infection, need to be investigated and treated. Prior to surgical intervention, a multidisciplinary team discussion should occur to facilitate streamlining of surgical plans and to devise a suitable anesthetic plan. The ANA team should be cognizant of the various surgical steps, required positioning, and effects on airway management throughout the case so as to be prepared for any potential complications.
